# Mechanistic diversity in MHC class I antigen recognition

**DOI:** 10.1042/BCJ20200910

**Published:** 2021-12-23

**Authors:** Camila R. R. Barbosa, Justin Barton, Adrian J. Shepherd, Michele Mishto

**Affiliations:** 1 Centre for Inflammation Biology and Cancer Immunology (CIBCI) & Peter Gorer Department of Immunobiology, King's College London, SE1 1UL London, U.K.; 2 Francis Crick Institute, NW1 1AT London, U.K.; 3 Department of Biological Sciences and Institute of Structural and Molecular Biology, Birkbeck, University of London, WC1E 7HX London, U.K.

**Keywords:** antigens, MHC class I, spliced peptides, T cells, TCR

## Abstract

Throughout its evolution, the human immune system has developed a plethora of strategies to diversify the antigenic peptide sequences that can be targeted by the CD8^+^ T cell response against pathogens and aberrations of self. Here we provide a general overview of the mechanisms that lead to the diversity of antigens presented by MHC class I complexes and their recognition by CD8^+^ T cells, together with a more detailed analysis of recent progress in two important areas that are highly controversial: the prevalence and immunological relevance of unconventional antigen peptides; and cross-recognition of antigenic peptides by the T cell receptors of CD8^+^ T cells.

## Immune response in cancer and infectious diseases

The immune response to infectious diseases or cancer shares similar systematic properties: the immune system can detect ‘non-self ‘ antigens that occur in both infected and tumour cells; it encompasses effector functions to specifically target and destroy the infected or tumour cells while protecting the host; it develops immunological memory via the adaptive immune responses that facilitates subsequent rapid defence mechanisms [1].

There are two different categories of immune response that afford protection and maintaining the host's normal state of homeostatic state: innate and adaptive. Immediate and generic immune responses are classified as innate due to their fast-acting nonspecific response against foreign antigens such as allergenic antigens, or non-self proteins and molecules [2]. The key players in cell-mediated innate immune responses are phagocytes and natural killer (NK) cells. These phagocytes (neutrophils, monocytes, and macrophages) facilitate immediate host protection by engulfing cells that express non-self-antigens or altered self-antigens and killing them with lysosomal enzymes [3].

In common with innate immunity, the adaptive immune response involves several components that can eliminate both pathogen-infected cells and tumour cells. Adaptive immunity can target antigens in infected and tumour cells by exploiting the effector functions of antibodies, T cells, B cells, and professional antigen-presenting cells (APCs) [2]. In general, endogenous antigens derived from intracellular bacteria, viruses and tumour cells are presented by Major Histocompatibility Complex class I (MHC-I) molecules at the cell surface and recognised by the T cell receptor (TCR) αβ heterodimers expressed at the cell surface of CD8^+^ T cells. Most cells express MHC-I molecules, and the reduced or null expression of MHC-I complexes at the cell surface can be patrolled by NK cells. Conversely, MHC-II molecules usually present exogenous peptides to TCRαβ of CD4^+^ T cells. MHC-II expression is usually limited to professional APCs [4].

## CD8^+^ T cell-mediated immunity and MHC-I antigen processing and presentation (APP) pathway

To participate in the adaptive immune response, a naive CD8^+^ T cell must first encounter an antigen, engage with professional APCs, and then be induced to differentiate into effector cells capable of removing the cells presenting the antigens, such as infected or tumour cells. In addition to providing effector T cells, a primary CD8^+^ T cell response generates immunological memory, giving protection from subsequent challenge by the same pathogen. This latter process is exploited in prophylactic vaccine development for infectious diseases [5] and therapeutic cancer vaccines targeting specific tumour-associated antigens [6,7].

CD8^+^ Cytotoxic T Lymphocytes (CTL) are a subset of T cells that have a crucial role in killing host cells infected with viruses, intracellular bacteria, and protozoans such as malaria [8–10]. In the anti-tumour immune response, CTLs such as CD8^+^ tumour-infiltrating lymphocytes (TILs) may make a pivotal contribution to the killing of MHC-I expressing tumour cells [11]. Although antibodies can recognise linear epitopes, they mainly recognise conformational epitopes exposed on the surfaces of proteins with tertiary structure. In contrast, CD8^+^ T cells recognise linear peptide fragments derived from the antigen, which are presented in an extended conformation. These antigenic peptides are presented by MHC-I molecules, and are preferentially 8–15 amino acid residues in length [12]. These peptides are used to discriminate non-self from self antigens [13], although there is a partial overlap between the pool of peptides derived from self- and non-self-antigens. We named those peptides that can be derived from both pathogens and from self as self/non-self *zwitter* antigenic peptides. *Zwitter* is the German word for ‘hybrid’ and ‘hermaphrodites’, originating from *zwi*-, meaning ‘duplex’ [14–16]. The real frequency of these peptides still has to be clarified, although various groups have made *in silico* estimates of self/non-self *zwitter* antigenic peptide frequency, taking into account the origins of unconventional antigenic peptide origins [15–23]. It has been hypothesised that they are involved in creating ‘holes’ in the T cell repertoire, involving mechanisms of central and peripheral tolerance, and in triggering autoimmune responses upon pathogen infection (see also [15,16] for details).

**Figure 1.**
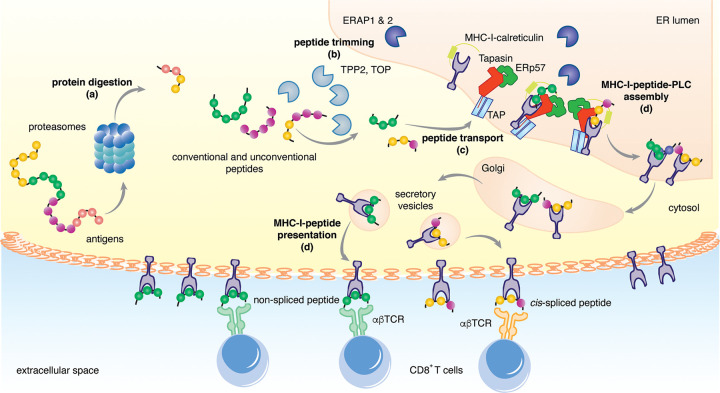
Key steps in the diversity of peptides presented via the MHC-I antigen processing and presentation (APP) pathway. The MHC-I APP pathway consists of a sequence of steps: (**a**) peptide generation mostly by proteasomes; (**b**) peptide trimming by aminopeptidases in the cytosol (e.g. TPP2) and in the ER (e.g. ERAP1); (**c**) peptide transport into the ER, mostly via TAP; (**d**) assembly of the MHC-I-peptide-PLC in the ER; (**e**) MHC-I-peptide presentation at the cell surface. All these steps modulate MHC-I immunopeptidome diversity.

The generation and loading of self and non-self peptides onto MHC-I molecules is carried out by the antigen processing and presentation (APP) machinery, and involves multiple steps: (a) peptide generation by proteasomes and other proteases; (b) peptide trimming by aminopeptidases; (c) peptide transport into the endoplasmic reticulum (ER); (d) assembly of the MHC-I-peptide loading complex (PLC) in the ER; and (e) MHC-I-peptide presentation at the cell surface, with the set of presented peptides known collectively MHC-I immunopeptidome [24] (Figure 1).

Most of the APP steps impinge upon the potential variety of peptide sequences in MHC-I immunopeptidomes by selecting which peptides are successfully presented. All molecules involved in MHC-I APP pathways can occur in different isoforms with different peptide sequence preference. Variation of the metabolic status of a cell, epigenetic regulation of gene expression and other cellular and molecular events can strongly affect the function and expression of all these molecules, thereby leading to a large diversity of MHC-I immunopeptidomes derived from cells of the same human being. Most of the molecules that are involved in the MHC-I APP pathway — e.g. proteasomes, ER aminopeptidases (ERAPs) and MHC-I — can also carry different polymorphisms or mutations that may strongly influence their activity [25–29]. In addition, some of the MHC-I APP steps can modify the original peptide sequence by ligating non-contiguous peptide fragments (peptide splicing) or modifying single amino acids through post-translational modifications (PTMs; e.g. oxidation, glycosylation, etc.), thereby generating antigenic peptides capable of being recognised by CTLs that cannot recognise the ‘parent’ sequence(s) from which that peptide was derived (Figure 1).

**Figure 2.**
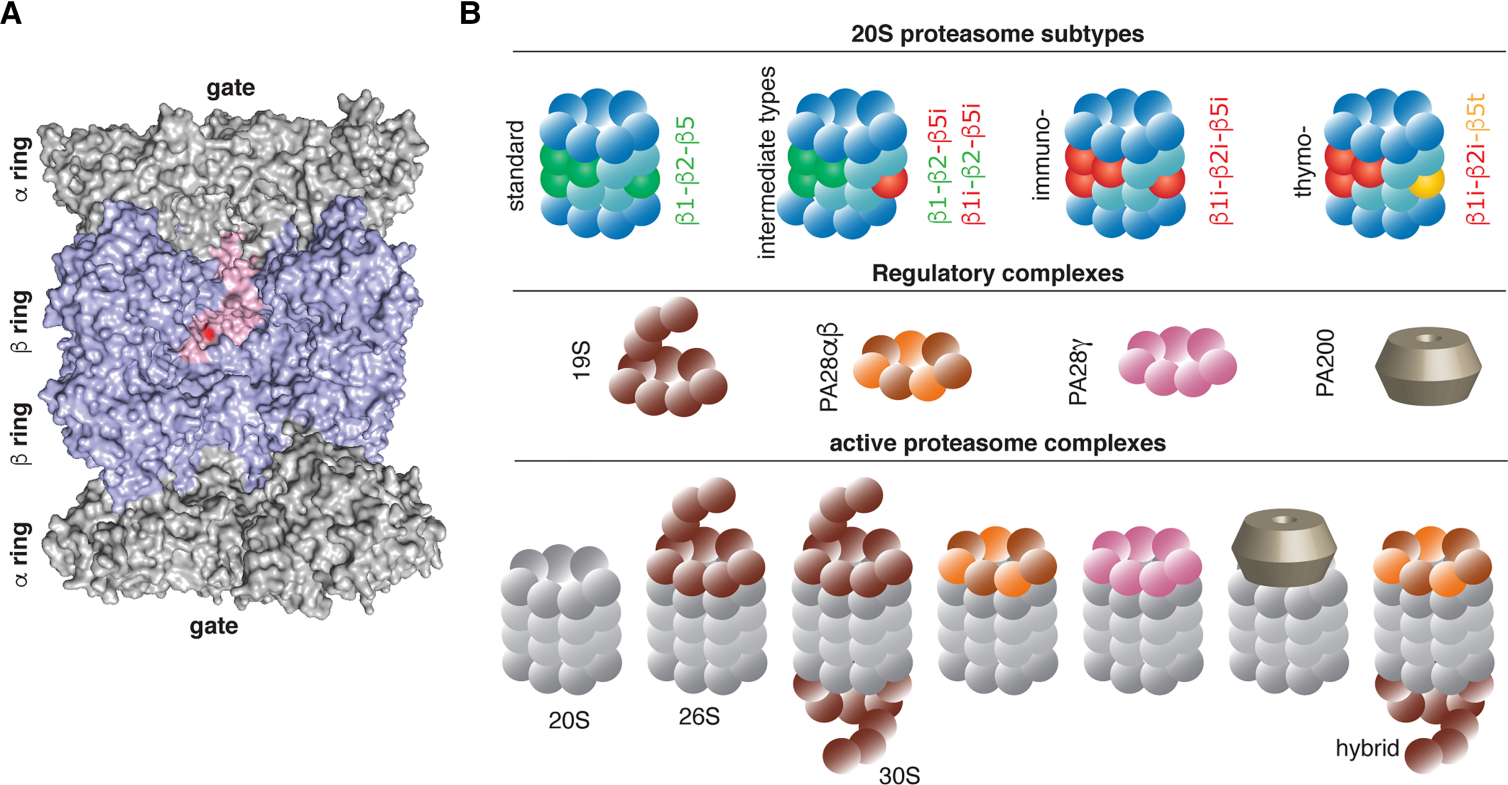
Proteasome isoforms. (**A**) The human 20S proteasome is shown based on the structure generated by Schrader et al. [30]. The chains B, C, H, I, J, Q, R, S, Y and Z are hidden from the structure to show the inner proteasome cavities with the central chamber and its two antechambers. The α and β subunits are coloured in grey and blue, respectively. The β2 subunit is shown in pink with its active site Thr_1_ in red, as an example of a catalytic subunit. (**B**) Proteasome isoforms. 20S proteasomes can be present in many isoforms, which vary on the base of their catalytic subunits. 20S proteasomes can binds to several regulatory complexes, which change both the conformation and the activity of the 20S proteasome core.

The starting point for the MHC-I APP pathway and the generation of MHC-I immunopeptidome diversity is the availability and selection of protein and polypeptide substrates. They can vary from cell to cell and over time within the same cell because of variations in cell metabolism, in the cell cycle, and in the activity of the ubiquitin–proteasome system (UPS), which is the main proteolytic pathway of cytoplasmatic proteins. In eukaryotic cells, the complete inhibition of proteasome activity causes cell death through necrosis and apoptosis. The multiple subunit 20S proteasomes are a pool of proteasome isoforms that are all associated with a common structure, characterised by two external α rings and two internal β rings. Each ring contains seven subunits. Each β ring contains three catalytic subunits, characterised by a Thr_1_ as active site [30] (Figure 2A). 20S standard proteasomes contain catalytic subunits β1, β2 and β5, whereas immunoproteasomes contain β1i/LMP2, β2i/MECL1 and β5i/LMP7 subunits, and thymoproteasomes contain β1i, β2i and β5t subunits (Figure 2B). Immunoproteasomes are expressed in immune cells such as mature dendritic cells (DCs), medullary thymic epithelial cells (mTECs) and in cells stimulated by IFN-γ and other inflammatory stimuli. Thymoproteasomes are almost exclusively expressed in cortical TECs (cTECs). There are also intermediate-type 20S proteasomes, which carry a different combination of standard-, immuno- and thymo-subunits [31]. Of note, cells generally contain a mixture of different 20S proteasome isoforms.

The effects of changes in the catalytic subunit composition of proteasome isoforms have been studied using a wide variety of methods. Modifications to the catalytic subunit composition alter the catalytic pocket and thus the substrate sequence preferences, and also induce subtle changes in proteasome conformation that affect its proteolytic dynamics [32,33]. All 20S proteasome isoforms can cleave after any substrate amino acid residue, but with very different frequencies, which greatly impacts the degradation rate of specific proteins and the amount of antigenic peptide produced [34–38]. These differences are key factors underpinning MHC-I immunopeptidome diversity, as proteasome isoforms can affect both cell metabolism — and thus what proteins are processed by UPS — and directly the antigenic peptides produced [39–45].

Although a proteolytic active role has been ascribed to 20S proteasomes in isolation under various conditions [46,47], this protease is often associated with regulatory particles such as 19S, PA28αβ, PA28γ and PA200, which affect proteasome conformation and activity [31,48–50] (Figure 2B). In 26S proteasomes, formed from the complex of 20S and the 19S regulator, the latter activates the 20S core, and binds to and unfolds ubiquitylated substrates. Proteins are poly-ubiquitylated through a cascade of E1, E2 and E3 enzymes, which activates, conjugates and transfers multiple ubiquitin moieties to protein substrates for degradation [51]. The other well-studied regulatory complex, PA28αβ, binds both standard- and immuno-proteasomes, although it has been hypothesised that it is more frequently associated with immunoproteasomes. The PA28αβ-20S proteasome complexes can degrade non-polyubiquitinated proteins [52,53].

Which regulatory complexes are bound to 20S proteasomes affects the features and sequences of antigenic peptides that are produced by the MHC-I APP pathway [54–58].

It is also worth noting that proteasomes are not the only proteases that generate peptides presented by MHC-I molecules, and some scientists have hypothesised that proteasomes make a less significant contribution to MHC-I APP than is generally supposed [59].

The second step of the MHC-I APP pathway that leads to diversity in the MHC-I immunopeptidomes and the presentation of epitopes is the trimming of peptides by amino peptidases. This can occur in the cytosol — e.g. by tripeptidyl peptidase 2 (TPP2) — as well as in the ER by ERAP1 and ERAP2. Aminopeptidases can shorten peptides, thereby generating the minimal epitopes that can be recognised by CD8^+^ T cells, as well as destroying potential antigenic peptides [60–62]. TPP2 and neurolysin can cleave large peptide fragments. Smaller peptides of 8–15 amino acids are handled by thymet oligopeptidase (TOP), and even smaller ones by other peptidases [63]. Binding of peptides longer than 8–9 amino acids, but not shorter ones, triggers a confirmational change in ERAP1 that activates its hydrolysis [64]. ERAP1/2 polymorphisms, such as the ERAP1 Lys/Arg528 polymorphism, can affect MHC-I immunopeptidomes, the diversity of the response against infections and the onset of autoimmune responses such as Ankylosing Spondylitis [29,65–69].

The third step of the MHC-I APP pathway that contributes to diversity in the MHC-I immunopeptidomes involves the transportation of peptides into the ER. Peptides have a half-life of 6–10 s in the cytosol of living cells, therefore the intracellular trafficking of peptides from the cytosol into the ER is responsible for the selection of peptides matching in length and sequence specificity to the respective MHC-I molecule [63]. The transporter associated with antigen processing (TAP) heterocomplex — formed by TAP1 and TAP2 — is a key molecule in this process [70]. Peptides with a length of 8–16 amino acids are preferentially transported by TAP, although longer peptides can also be transported by TAP, but with less efficiency [71]. The TAP complex has peptide sequence preferences, which may reduce the transportation of ‘optimal’ epitopes in favour of their N-extended precursors [72,73]. The TAP-independent transport of peptides into the ER has also been described [62,63,74]. TAP forms a molecular complex with other proteins of the peptide loading complex (PLC) such as the oxidoreductase ERp57, the MHC-I heterodimer, and the chaperones tapasin and calreticulin [75]. The latter hold the empty MHC-I complexes in a peptide-receptive state, and tapasin promotes MHC-I-peptide binding with a slow off rate, thereby helping to shape MHC-I immunopeptidomes [64].

The last step of the MHC-I APP pathway that affects MHC-I immunopeptidome diversity, which we consider in this review, is the binding of peptides to the complex that presents antigenic peptides to the TCRs of CD8^+^ T cells, i.e. the MHC-I complex. The binding affinity and stability of the complex formed between the MHC-I complex and an antigenic peptide depends on two factors. First, the peptide that binds to MHC-I molecules forms a hydrogen bonding network that is conserved in the N- and C- terminal regions of the peptide and the corresponding heavy chain residues of the MHC-I, namely those associated with pockets A and F [76]. The interaction between the peptide's N- and C-termini with the MHC-I groove contributes more to the binding energy of the MHC-I-peptide complexes than interactions involving the peptide side chains [77–79]. This is regarded as the main limiting factor in terms of the length of the peptides presented by MHC-I molecules, which is a predominantly in the range 9 to 12 amino acids in humans and 8 to10 amino acids in mice, although longer peptides can also bind to MHC-I complexes with lower frequency [80–87]. Second, the MHC-I binding groove has two (or less commonly three) pockets that show a preference for one to five (more often one or two) of the 20 possible amino acid side chains [88]. One of these pockets always accommodates the C-terminus of the peptide. The residues lodged by the other pocket vary, depending on the shape, but are almost always the second, third or fifth N-terminal residue of the peptide [89]. In human beings, MHC-I heavy chains are encoded by three genes (*HLA-A, -B and -C*) located in chromosome 6. These genes are the most polymorphic genes in the human genome. The allelic variation mainly affects the nature and composition of the peptide-binding groove and thus modulate MHC-I immunopeptidomes [76].

## Conventional and unconventional antigenic peptides

**Figure 3.**
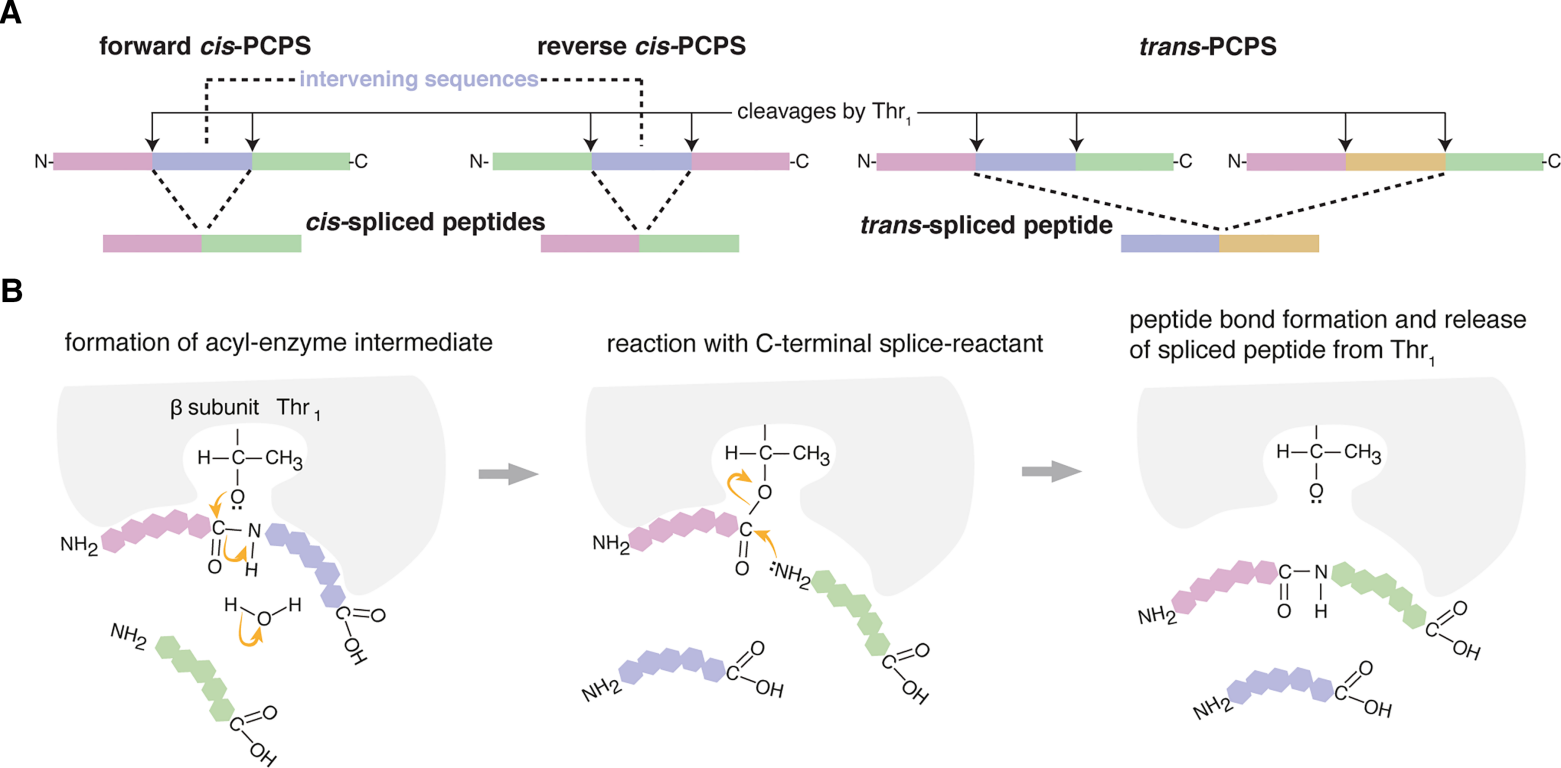
Proteasome-catalysed peptide splicing (PCPS). (**A**) Spliced peptides can be formed by: (i) *cis-*PCPS, i.e. when the two splice-reactants derive from the same protein. The ligation of the splice-reactants can occur in normal order, i.e. following the orientation from N- to C-terminus of the parental protein (forward *cis*-PCPS), or in the reverse order (reverse *cis*-PCPS); (ii) *trans*-PCPS, when the two splice-reactants originate from two distinct proteins. (**B**) Transpeptidation. Proteasome's catalytic Thr_1_ breaks the peptide bond of the residue (P1) of the protein - thereby forming an acyl-enzyme intermediate with the N-terminal splice-reactant, and releasing the C-terminal peptide fragment. The acyl-enzyme intermediate can then interact with another peptide fragment (the C-terminal splice-reactant) and form a new peptide bond between the P1 residue of the N-terminal splice-reactant and the residue P1′ of the C-terminal splice-reactant.

One of the mechanisms underpinning MHC-I immunopeptidome diversity is the generation of antigenic peptides that are not derived straightforwardly via the translation of canonical exon sequences. These unconventional peptides were substantially overlooked until the beginning of the millennium and their discovery is gaining momentum through the continuous development of novel methods for their identification. A sizable portion of these peptides is represented by peptide sequences that derive from putative non-coding regions of the genomes, such as 5′- and 3′-UTRs, introns, intergenic and endogenous retroviral regions [90–96]. We also have peptide sequences that derived from non-canonical open reading frames (ORFs), frameshifted canonical genes, and alternative RNA splicing [97–99]. Other sources of unconventional antigenic peptides are protein fragments that contain single amino acid polymorphisms or somatic mutations. The former are examples of the minor histocompatibility antigens and play a role in organ transplantation. The latter, which are known as neoepitopes when they trigger a CTL response, are particularly relevant in cancer and are proving to be attractive targets for immunotherapies [100–105]. All these are unconventional peptides that can be produced by proteasomes via peptide hydrolysis and do not require any PTM. In addition, peptides may either acquire PTMs — e.g. via phosphorylation [106–109] — or the original antigen sequences may be reshuffled by the ligation of non-contiguous peptide fragments. This latter process, called peptide splicing, has been described in the context of both MHC-I and -II pathways. In the former, the process is mainly catalysed by proteasomes, whereas in the latter, the role of lysosomal proteases such as cathepsin L has been hypothesised and can explain the production of hybrid insulin peptides [110–112]. Proteasome-catalysed peptide splicing (PCPS) can occur by combining two fragments of the same protein (*cis*-PCPS) or two distinct protein molecules (*trans*-PCPS; see Figure 3A). The main biochemical mechanism of PCPS is transpeptidation, as proposed by Vigneron et al. [113], in the first study describing PCPS in the context of tumour. During transpeptidation, a peptide is cleaved. The N-terminal fragment forms an acyl-enzyme intermediate with Thr_1_ of a proteasome catalytic subunit. This intermediate can interact with water and then be released as canonical non-spliced peptide by peptide hydrolysis. Alternatively, the acyl-enzyme intermediate can interact with another peptide fragment and form a spliced peptide through PCPS (Figure 3B). PCPS via transpeptidation has been demonstrated using various biochemical and cellular biology strategies [114–117]. A single example of condensation reaction has also been described as an alternative PCPS mechanism [118].

First discovered by Van den Eynde et al. [113,119], proteasome-generated spliced peptides have since proved controversial. In the last decade, one common opinion (to quote a reviewer of a grant application rejected a decade ago) was that peptide splicing is ‘a rare event and a curiosity of nature’, although this opinion was not supported by experimental evidence. The subsequent identification, in MHC-I immunopeptidomes, of *cis*-spliced peptides by Mishto et al. [81], and *trans*-spliced peptides by Faridi, Croft, Purcell and colleagues [80] has served to heighten the controversy. Both of these studies have been intensively scrutinised by other groups working on immunopeptidomics, and the ensuing disagreements have left many in the scientific community uncertain about the true frequency of spliced peptides in MHC-I immunopeptidomes [120–123]. This controversy spanned the fields of mass spectrometry and immunopeptidomics — perhaps nurtured by the same general scepticism that welcomed the pioneering research on PCPS — and matured in the hypothesis that PCPS ‘is, at most, an extremely rare event and likely does not happen at all’ [120]. As discussed in a recent commentary [124], this hypothesis clashes with a good deal of experimental evidence demonstrating that proteasomes can efficiently catalyse both peptide hydrolysis and splicing. For instance, *cis-*spliced epitopes can trigger a specific CD8^+^ T cell response against tumour-associated [40,113,117–119,125,126], Type1 Diabetes-associated [99] and *Listeria monocytogenes*-derived [127] antigens. *Cis-*spliced epitopes can also stimulate CTL cross-recognition during infections through TCR degeneracy (see below) [128,129]. Successful application of immunotherapies targeting *cis*-spliced epitopes has been described in the treatment of a metastatic melanoma patient using an autologous tumour-infiltrating lymphocyte clone [125,130], in nonobese diabetic/severe combined immunodeficient mice leading to engraftment inhibition of human acute myelogenous leukaemia cells [117,131], as well as in a mouse model of glioblastoma via peptide vaccination [132]. Although *cis*-PCPS might theoretically generate a very large number of peptide sequences that, preliminary estimates suggest it has a limited role in T cell tolerance and viral-driven autoimmunity [15,16]. The biochemical aspect of PCPS can be studied in relatively simple experiments, wherein synthetic polypeptides are processed by purified proteasome isoforms, and their products are detected via mass spectrometry. The largest database of non-spliced and spliced peptides produced by proteasomes in these *in vitro* assays has been published by Specht and colleagues [133], and contains almost 15 000 unique peptides. In this database, *cis*-spliced and *trans*-spliced peptides represent each one third of the peptide product variety [133], although this experimental set up might artificially favour PCPS [124]. These results seem to confirm that *cis*-spliced peptides may make a substantial contribution to MHC-I diversity, as suggested by *in cellula* experiments [40,80,132,134]. However, unbiased evaluation of all unconventional antigenic peptides in relation to conventional peptides still needs to be carried out, and may ultimately resolve current disputes about this topic.

## T cell receptor (TCR)

**Figure 4.**
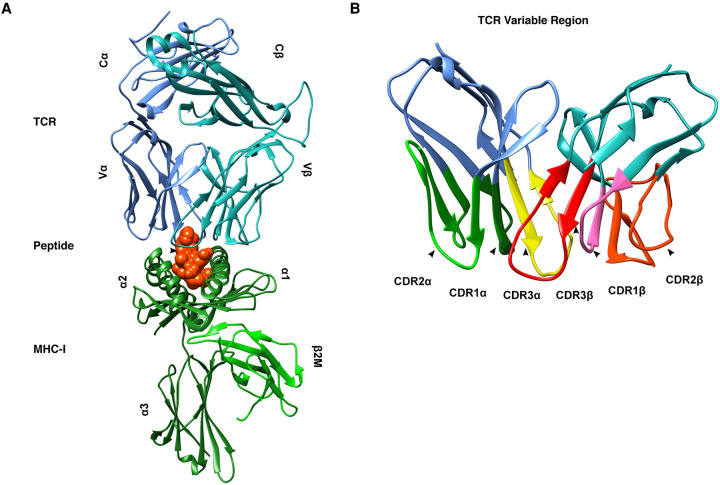
Structure of a CD8^+^ TCR. (**A**) A CD8^+^ TCR bound to a cognate MHC-I-peptide complex. The TCR is shown in blue (α chain) and cyan (β chain) with the constant (C) and variable (V) regions annotated. The peptide is shown in red and the MHC-I in green with the α-helices (α1 through α3) and β2 microglobulin (β2M) annotated. (**B**) Looking more closely at the variable region of the TCR reveals the six complementarity determining regions (CDRs) at the interface with the MHC-I-peptide complex. Note the central position of the CDR3s on both the α and β chains; these are the CDRs most associated with peptide contacts.

Whereas the MHC-I APP pathway can be viewed as the cornerstone of the MHC-I immunopeptidomes diversity, TCRs expressed on the surface of CD8^+^ T cells arguably play a comparable role with respect to the diversity of antigen recognition. Those TCRs that recognise MHC-I-peptide complexes are heterodimeric proteins, composed of an α chain and a β chain (Figure 4A). Other types of TCRs with different function and specificities have also been described [135]. Each α and β chain have three protruding loops that contact the MHC-I-peptide complex (Figure 4B). Owing to their role in binding the MHC-I-peptide complex, these six loops are referred to as complementarity determining regions (CDRs). Of these six CDRs, analyses of solved crystal structures suggest that the third loop on the β chain, referred to as CDR3β, has the greatest impact on the interaction of TCR and MHC-I-peptide complex, and the antigenic peptide in particular [136].

TCR sequences are generated by recombination of variable (V), diversity (D), and joining (J) gene segments during T cell maturation in the thymus. This VDJ recombination process leads to a vast space of possible TCRs, with estimates of 10^15^ distinct possible combinations [137,138]. Of course, not all possible combinations are viable nor are all viable combinations observed with equal frequency. Recombinatorial biases arise where proximal sites in some loci undergo recombination much more frequently than distal sites. This leads to biased and constrained TCR ‘repertoires’ [139]. The set of TCRs within an individual subject is referred to as the TCR repertoire. The TCR repertoire is refined by a selection process that promotes useful TCRs that successfully bind antigens and down-regulates potentially harmful TCRs which bind self-antigens. This selection process is often referred to as ‘thymic selection’ since it takes place in the thymus. The TCR repertoire is thus shaped by the history of pathogens that a host has been exposed to [140].

Given the apparent primacy of CDR3β in determining the specificity of TCR binding with respect to a given antigenic peptide, many popular algorithms have been developed which cluster TCRs based on the sequence similarity of the CDR3β region [141–144]. These algorithms purport to identify clusters of TCRs with similar antigen specificity. More recently, there has been evidence suggesting that the CDRs most actively involved in MHC-I-peptide binding can vary by MHC-I-peptide complexes. This is likely due to the complex conformational changes in both the TCR and the MHC-I-peptide complex that can facilitate different binding arrangements [145,146]. This conformational plasticity is one mechanism by which a single TCR can potentially recognise many different antigen-derived peptides.

## TCR cross-reactivity

A single TCR can potentially recognise more than a million different peptides [147]. Conversely, a peptide can elicit a response from millions of TCRs [138]. This flexibility is referred to as ‘cross-reactivity’, or ‘degeneracy’.

The number of peptides which can be presented in MHC-I-peptide complexes is estimated to be greater than 10^15^ [138]. This estimate is derived by calculating all the possible combinatorial permutations of the 20 common amino acids for peptide lengths that can be presented by MHC molecules (8 to 14 residues), then estimating that 1% to 3% of these possible peptides will bind with and be presented by MHC molecules. On the other hand, the number of unique TCRs within an individual's repertoire is estimated to be in the range of 10^6^ [148] to 10^8^ [149]. This indicates that an individual's ability to mount a successful immune response to a broad proportion of the potential pathogen space is partially reliant on cross-reactivity, as it facilitates a broader range of specificity than would otherwise be possible within the physical constraints of the adaptive immune system. In the context of the vast universe of presented peptides, however, even a highly cross-reactive TCR only recognises a tiny fraction. Ishizuka et al. [19] empirically estimated the likelihood of unrelated peptides being recognised by the same TCR as 1 in 30 000.

Cross-reactivity comes at a cost, however. Cross-reactivity can mean that response to a particular antigen has off-target effects, as is often the case in autoimmunity and allergy. Unanticipated cross-reactivity is a major concern, since on-target and off-target toxicity can be extremely damaging, and even fatal. On-target toxicity occurs when introduction of an antigen causes unintentional cytotoxic destruction of healthy tissues. Off-target toxicity occurs in situations such as molecular mimicry where different antigens are presented as MHC-I-peptide complexes with similar key structural and chemical features and bind with the same TCRs, potentially leading to unintended collateral damage [150]. The self-non-self *zwitter* epitopes described above are a particular example of cross-recognition. Indeed, both self-derived and non-self-derived *zwitter* epitopes can be recognised by the same TCR, since they have exactly the same peptide sequence.

A corollary of cross-reactivity is what is called ‘heterologous immunity’. Heterologous immunity occurs when previous infection by one pathogen confers immunity against an unrelated pathogen. It is important to note that heterologous immunity is not always bi-directional. For example, infection with Influenza A in mice confers protection against Vaccinia, but the reverse is not true [151].

As one might expect, instances of cross-reactivity have been observed between peptides with a high degree of sequence similarity. Perhaps less intuitively, instances have also been observed of peptides which bind to the same TCR but share almost no sequence similarity [152].

**Figure 5.**
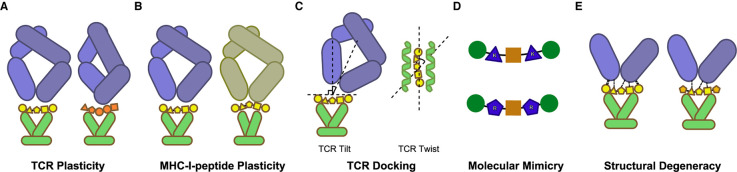
Structural mechanisms of T cell cross-reactivity. (**A**) Cross-reactivity can be facilitated by the significant conformational plasticity of the TCR, particularly in the CDR loops. Here a TCR (blue) changes conformation to bind different MHC-I-peptide complexes. (**B**) Similarly, the MHC-I-peptide complex can undergo conformational changes that promote binding with different TCRs. (**C**) Variable TCR docking angles can alter the interface and allow for binding a broader range of MHC-I-peptide complexes. TCR docking angles vary substantially in the vertical (TCR tilt) and horizontal (TCR twist) planes. (**D**) Different peptides can bind with the same TCRs by presenting very similar physicochemical surfaces at the interface. Here peptides are shown substituting arginine (R) residues for lysine (K) residues, which conserve physicochemical features such as positive charge and polarity. (**E**) TCR-MHC-I-peptide binding is an interplay of multiple factors including shape complementarities, hydrogen bonds, salt bridges, and van der Waals interactions. This confederation of forces includes some redundancy and often binding can be achieved with a subset of the factors. This allows binding to be resilient to substitutions in peptide sequences.

Cross-reactivity is driven in large part by conformational flexibility in the TCR, peptide, and MHC, leading to a high diversity in the interfaces between the TCR and MHC-I-peptide complexes [153]. These different interfaces can have very different contact residues and conformational geometries. There are five known mechanisms that can lead to these varied interfaces: TCR plasticity, MHC-I-peptide complex plasticity, TCR docking, molecular mimicry, and structural degeneracy [154] (Figure 5).

Perhaps the most broadly discussed of these mechanisms is the conformational plasticity of the TCR, particularly in the CDR loops (Figure 5A). This flexibility allows the TCR to accommodate different MHC-I-peptide complexes without changing the docking orientation. Several studies have observed and quantified just how variable the conformations of the CDR loops can be. Distances between the bound and unbound states typically range from 0.3 Å to 11.4 Å with the CDR3 loop exhibiting the greatest range of motion [155].

In a similar fashion, MHC-I-peptide complexes can exhibit varying degrees of conformational flexibility, which can likewise allow for substantial rearrangement at the time of TCR binding [154] (Figure 5B). At the present time this mechanism has been less studied and is less well-understood, though anecdotal examples are presented in [156,157]. Riley et al. [158] reported a particularly interesting example of unexpected cross-reactivity between a TCR and peptides sharing few physiochemical commonalities. Binding with the TCR induced substantial conformational changes in one MHC-I-peptide complex, including a register shift that resulted in a 3.5 Å root-mean-square deviation (RMSD) movement for all peptide atoms. A ‘register shift’ refers to a shift in the peptide such that residues lie in different positions within the MHC groove, potentially resulting in attendant changes in peptide bulges and side chain orientations.

Variable docking orientation is another mechanism by which a TCR can bind to different MHC-I-peptide complexes (Figure 5C). These docking angles can be quite variable in both the vertical (referred to as ‘TCR tilt’) and horizontal (referred to as ‘TCR twist’) planes. To-date incident angles (measuring TCR tilt) have mostly been observed to fall within the range 0° to 30° range (relative to the MHC normal vector) and crossing angles (measuring TCR twist) within the range 22° to 69° range (relative to the MHC groove vector) though outliers have been observed substantially outside of these ranges and the sample size of solved structures remains relatively small [157,159]. The TCR3d database [160] provides known TCR structures including their docking angles.

‘Molecular mimicry’ refers to instances where MHC-I-peptide complexes that are unrelated can share key structural and chemical features, thereby allowing a TCR to recognise both complexes (Figure 5D). It has been demonstrated that even peptides with low sequence and physicochemical similarity can present very similar MHC-I-peptide surfaces at their interface with TCRs. In an extreme case, Zhang et al. [161] presented an example of cross-reactivity between peptides with no sequence overlap. Cross-reactivity driven by molecular mimicry has been successfully predicted using machine learning approaches trained on feature data including surface electrostatic charges and accessible surface area of selected residues in MHC-I-peptide complexes [152,162], at least for a specific MHC-I allele (HLA-A*02:01).

In some cases, TCRs can bind to MHC-I-peptide complexes with only a subset of the binding mechanisms that might otherwise be present, including some cases with no hydrogen bonds or salt bridges and poor shape complementarity (Figure 5E). This degeneracy allows TCR binding to be resilient to substitutions in the contact residues of related peptides.

Note that the mechanisms of TCR cross-reactivity discussed here are laid out for conceptual understanding and are by no means mutually exclusive. Combinations of these mechanisms have been observed and it is likely that cross-reactivity dynamics include a complex interplay of various mechanisms. Note also that peptide recognition is not a binary event, and affinity can vary substantially. At the interface between the TCR and MHC-I-peptide complexes, several residues may participate in binding, influencing the affinity and the specific level of T-cell stimulation that will be triggered by each MHC-I-peptide complex. Due to limitations in current experimental methods, available data may be biased towards higher-affinity interactions as low-affinity interactions can escape detection.

Though binding affinity is an important consideration, it is worth noting that activation is not always correlated with affinity. In fact, TCR-MHC-I-peptide complexes with high binding affinity but no detectable T cell activation occur frequently *in vivo* in humans [163]. Activation is also not strictly a binary event, as the consequence of T cell activation can vary, for example with respect to differences in cytokine production.

Cross-reactivity is a key component in the functional dynamics of adaptive immunity. It has direct implications for immunotherapies, autoimmunity, and heterologous immune response. As immunotherapies grow in popularity, the need to reliably predict cross-reactivity will be key to safe and effective interventions.

## Our limited knowledge and future directions

In conclusion, there has been a giant leap in our understanding of the mechanistic diversity of MHC-I antigen presentation and recognition during recent decades. However, the journey is still long, and areas that are still neglected and controversial, such as the presence and features of unconventional epitopes and the drivers of TCR degeneracy, may represent important directions for future research, and contribute to the development of novel immunotherapies against cancer and pathogens.

## Abbreviations

APC: antigen-presenting cells
APP: antigen processing and presentation
CDR: complementarity determining regions
cTECs: cortical thymic epithelial cells
CTL: CD8^+^ Cytotoxic T Lymphocytes
DC: dendritic cells
ER: endoplasmic reticulum
ERAP: ER aminopeptidases
ERp57: endoplasmic reticulum resident protein 57
LMP2: low-molecular mass polypeptide 2
LMP7: low-molecular mass polypeptide 7
MECL-1: multicatalytic endopeptidase complex-like-1
MHC-I: Major Histocompatibility Complex class I
mTECs: medullary thymic epithelial cells
NK: natural killer
PCPS: proteasome-catalysed peptide splicing
PLC: peptide loading complex
PTM: post-translational modifications
RMSD: root-mean-square deviation
TAP: transporter associated with antigen processing
TCR: T cell receptor
TILs: tumour-infiltrating lymphocytes
TOP: thymet oligopeptidase
TPP2: tripeptidyl peptidase 2
UPS: ubiquitin–proteasome system
β2M: β2 microglobulin
